# Diagnostic Utility of Muscle Ultrasound for Sarcopenia in Prader–Willi Syndrome: A Cross‐Sectional Study

**DOI:** 10.1002/jcsm.70326

**Published:** 2026-06-08

**Authors:** Luo‐Hua Yu, Hsin‐Chi Wu, I‐Shiang Tzeng, Yan‐Ting Luo, Li‐Ping Tsai, Valeria Chiu

**Affiliations:** ^1^ Department of Physical Medicine and Rehabilitation Taipei Tzu Chi Hospital, Buddhist Tzu Chi Medical Foundation New Taipei City Taiwan; ^2^ Department of Medicine Tzu Chi University Hualien Taiwan; ^3^ Department of Research Taipei Tzu Chi Hospital, Buddhist Tzu Chi Medical Foundation New Taipei City Taiwan; ^4^ Department of Pediatrics Heping Fuyou Branch, Taipei City Hospital Taipei Taiwan; ^5^ Department of Molecular Biology and Human Genetics, College of Medicine Tzu Chi University Hualien Taiwan

**Keywords:** body composition, Prader–Willi syndrome, sarcopenia, ultrasonography

## Abstract

**Background:**

Prader–Willi syndrome (PWS) is characterized by sarcopenic obesity; however, validated screening tools for muscle mass in clinical settings are lacking. This study aimed to evaluate the diagnostic utility of muscle ultrasound (US) for detecting low muscle mass in individuals with PWS.

**Methods:**

This observational, cross‐sectional study recruited 48 individuals with genetically confirmed PWS (International Classification of Diseases, 10th Revision Code Q87.1) from a specialized clinic (October 2022–June 2024). Appendicular skeletal muscle index (ASMI) via dual‐energy x‐ray absorptiometry (DXA) served as the primary outcome. Sarcopenia was defined based on Asian Working Group for Sarcopenia (AWGS) 2019 criteria. US predictors included muscle thickness (MT) of the rectus femoris (RF), vastus lateralis and gastrocnemius medialis (GM); pennation angle; and RF cross‐sectional area (CSA). Analysis included Spearman's correlation (ρ), multivariable linear regression (B) and receiver operating characteristic (ROC) curves.

**Results:**

The cohort (*n* = 48; 47.9% women; median age 19.5 years, interquartile range [IQR] 12.3–26.8) had a 100% prevalence of obesity (median BMI 27.1 kg/m^2^, IQR 22.2–31.4). All participants (100%) had a history of growth hormone treatment; 62.5% exhibited the deletion subtype. Low muscle mass was observed in 60.4% (*n* = 29), confirmed sarcopenia in 52.1% (*n* = 25) and severe sarcopenia (including low physical performance) in 20.8% (*n* = 10) participants. GM MT showed the strongest correlation with ASMI (ρ = 0.689, *p* < 0.001, 95% confidence interval [CI] 0.50–0.81), followed by RF CSA (ρ = 0.587, *p* < 0.001). Multivariable regression identified GM MT as a significant independent predictor of ASMI (B = 0.957, *p* = 0.011) after adjusting for BMI (B = 0.128, *p* < 0.001), age (B = 0.020, *p* = 0.044) and sex (B = −0.376, *p* = 0.029; *R* ^2^ = 0.67). ROC analysis for detecting low muscle mass yielded an area under the curve for GM MT of 0.759 (95% CI 0.616–0.901, *p* = 0.003), with an optimal cut‐off of 1.69 cm (sensitivity 86.2%, specificity 63.2%).

**Conclusions:**

Sarcopenia affects 52.1% of patients with PWS, with 20.8% meeting criteria for severe sarcopenia. Ultrasound of the gastrocnemius medialis is a valid, radiation‐free predictor of skeletal muscle mass and serves as a practical diagnostic tool for sarcopenic obesity in PWS.

## 1 Introduction

Prader–Willi syndrome (PWS) is a complex neurodevelopmental disorder resulting from the lack of expression of paternally derived genes on chromosome 15q11–q13 [1]. It is the most common genetic cause of life‐threatening obesity; however, the body composition phenotype of PWS extends beyond excess adiposity. Individuals with PWS exhibit a distinct sarcopenic profile characterized by reduced lean body mass and decreased muscle strength, which persists even in patients treated with growth hormone [2]. This coexistence of low muscle mass and high adiposity fits the diagnostic paradigm of sarcopenic obesity, a condition associated with greater metabolic, functional and cardiovascular risks than either obesity or sarcopenia alone [3].

Accurate assessment of skeletal muscle mass is therefore critical for the clinical management of PWS. dual‐energy x‐ray absorptiometry (DXA) is considered the gold standard for assessing appendicular skeletal muscle index (ASMI) in clinical practice [4]. However, DXA has strong limitations in routine monitoring, including ionizing radiation exposure, high cost and limited accessibility. Moreover, in the PWS population, severe obesity often presents physical barriers, because patients may exceed the weight capacity or scan field dimensions of the DXA table. Additionally, patients may present with behavioural difficulties during the procedure. Beyond physical barriers, DXA may also provide inaccurate readings in this population. Fintini et al. highlight that in children with obesity, who are often taller and have larger bones, DXA may overestimate true bone density because it generates a two‐dimensional image of a three‐dimensional structure [5]. Consequently, a pressing need remains for a non‐invasive, radiation‐free and accessible bedside tool to screen for sarcopenia and monitor the efficacy of therapeutic interventions in this population.

Muscle ultrasound has emerged as a reliable alternative to DXA for estimating muscle mass and diagnosing sarcopenia in the general geriatric and adult populations [6]. It offers the advantages of portability, lack of radiation and the ability to visualize muscle architecture parameters such as muscle thickness and pennation angle. The European Working Group on Sarcopenia in Older People (EWGSOP2) and the Asian Working Group for Sarcopenia (AWGS) have increasingly recognized ultrasound as a potential diagnostic tool [7, 8].

Despite its growing popularity, validated ultrasound protocols specific for the PWS population are lacking. Most existing sarcopenia studies focus on the rectus femoris of the thigh as the primary measurement site [9]. However, in individuals with PWS, the characteristic accumulation of subcutaneous adipose tissue in the thigh region may attenuate ultrasound signals and compromise measurement accuracy, a limitation previously described in general obesity populations [6]. Conversely, biomechanical studies suggest that the distal anti‐gravity muscles, such as the gastrocnemius, are often preserved or even hypertrophied in obese individuals due to the chronic loading of excess body weight during ambulation [10].

Therefore, the aim of this study was to (1) evaluate the correlation between muscle ultrasound parameters (thickness and pennation angle) and DXA‐derived muscle mass indices, in a cohort of patients with PWS; (2) compare the predictive utility of the thigh (rectus femoris/vastus lateralis) versus the calf (gastrocnemius medialis); and (3) establish optimal cut‐off values to facilitate the screening of low muscle mass in clinical practice. We hypothesized that due to the preserved ambulatory function often observed in patients with PWS, distal lower limb muscles (including the gastrocnemius) would be less affected by subcutaneous adiposity and thus serve as a more accurate predictor of skeletal muscle mass than the proximal thigh muscles.

## 2 Materials and Methods

### 2.1 Study Design, Setting and Duration

This observational, cross‐sectional study was conducted at a single‐centre specialized outpatient clinic for rare diseases at Taipei Tzu Chi Hospital, Taiwan. The study duration spanned October 2022 to June 2024. The study protocol adhered to the Strengthening the Reporting of Observational Studies in Epidemiology (STROBE) statement [11].

### 2.2 Participants and Eligibility Criteria

We recruited individuals aged 7 years and older with a genetically confirmed diagnosis of PWS (International Classification of Diseases, 10th Revision code Q87.1). Diagnosis and genetic subtype (deletion vs. non‐deletion) were confirmed by a board‐certified medical geneticist or paediatric endocrinologist with over 30 years of experience in rare disorders through methylation‐specific PCR, fluorescence in situ hybridization or chromosomal microarray analysis. To ensure study population homogeneity and control for potential confounding factors affecting muscle metrics, strict inclusion and exclusion criteria were applied. Inclusion criteria include (1) confirmed PWS diagnosis; (2) ambulatory status with sufficient physical and cognitive capacity to complete performance tests and follow instructions; and (3) current residence in Taiwan (to ensure environmental and dietary homogeneity). Exclusion criteria include (1) coexisting neuromuscular or orthopaedic disorders (e.g., severe joint arthritis, untreated scoliosis or musculoskeletal deformities) preventing accurate ultrasound or DXA positioning or physical performance testing; (2) history of lower limb surgery or fracture within the past 6 months; (3) current use of medications significantly altering muscle metabolism (e.g., systemic corticosteroids or anabolic steroids), except for the standard growth hormone therapy; and (4) acute illness or hospitalization within the previous 3 months to avoid confounding effects of acute catabolic states.

### 2.3 Sample Size Determination

Based on previous research in PWS reporting strong correlations (*r* = 0.79–0.89) between motor performance and quality of life [12], an a priori power analysis was conducted using G*Power (v3.1.9.4) [13]. Using a conservative large effect size (*r* = 0.50), a minimum sample size of 38 participants was required to achieve a statistical power (1 − *β*) of 0.90 at (*α*) of 0.05. Our final cohort of *n* = 48 exceeded this requirement, providing a post hoc power of > 0.99 for the primary correlation analyses between muscle ultrasound parameters and ASMI.

### 2.4 Clinical and Anthropometric Assessment

Demographic variables (age, sex and genetic type) were collected. Clinical comorbidities were documented based on physician diagnosis and current medication usage. Type 2 diabetes mellitus was defined according to the American Diabetes Association (ADA) criteria (fasting plasma glucose ≥ 126 mg/dL, 2‐h post‐prandial glucose ≥ 200 mg/dL or HbA1c ≥ 6.5%). Dyslipidaemia was defined based on the National Cholesterol Education Program (NCEP) Adult Treatment Panel III (ATP III) criteria (LDL‐C > 130 mg/dL) or the current use of lipid‐lowering agents. Anthropometric measurements included height, weight and body mass index (BMI).

### 2.5 Muscle Ultrasound Procedure and Measurements

Ultrasonography was performed using a LOGIQ S8 system (GE Healthcare, UK). A linear probe (6–15 MHz) was used for standard assessment, whereas a curved probe (1–6 MHz) was utilized when the muscle cross‐section exceeded the field of view. To ensure high intra‐rater reliability, all ultrasound assessments were performed by a single, board‐certified physiatrist with over 10 years of experience in musculoskeletal imaging. Generous amounts of coupling gel were applied to prevent tissue compression. Previous validation studies comparing ultrasound to magnetic resonance imaging have demonstrated excellent reliability (ICC > 0.90) and validity for muscle thickness and cross‐sectional area measurements in both healthy and clinical populations [6, 13].

Evaluation was performed in B‐mode in the transverse and longitudinal planes for the rectus femoris, vastus lateralis and gastrocnemius medialis of the right lower limb.

- Rectus femoris: Participants were positioned lying in a supine position with the arms and legs fully extended for muscle relaxation. The transducer was placed perpendicular to the long axis of the thigh at two specific points: (1) three‐fifths of the distance from the anterior superior iliac spine (ASIS) to the superior patellar border and (2) at the midpoint of this length [9, 13]. These points represent the maximal cross‐sectional area sites. RF MT was measured as the distance between the superficial and deep fascia. Rectus femoris cross‐sectional area (RF CSA) was measured using a planimetric technique by tracing the inner echogenic rim of the muscle fascia.
- Vastus lateralis: The probe was positioned laterally at the midpoint between the ASIS and the superolateral patellar border [9]. Vastus lateralis muscle thickness was measured in transverse images, and the pennation angle, the angle between muscle fibres and deep fascia, was measured in longitudinal images [13, 14].
- Gastrocnemius medialis: Participants laid in a prone position with the legs fully extended and feet hanging off the edge of the bed. Gastrocnemius medialis muscle thickness (GM MT) was measured along the mid‐sagittal line at the maximal girth point between the proximal and distal tendon insertions. GM MT and pennation angle were recorded [6, 9].

### 2.6 Body Composition and Physical Performance Assessment

Whole‐body composition was evaluated using a Hologic QDR‐4500A DXA system, a validated instrument for quantifying appendicular lean mass [15]. Skeletal muscle mass, ASMI and fat mass index were calculated. Sarcopenia and obesity thresholds were stratified by age to account for the paediatric and adult cohorts within our study population. In adults, low muscle mass was defined according to the AWGS 2019 guidelines as an ASMI < 7.0 kg/m^2^ in men and < 5.4 kg/m^2^ in women [8]. In children and adolescents, low muscle mass was defined as an ASMI Z‐score < −1 (more than one standard deviation below the mean) of the age‐ and sex‐specific reference population [16]. Obesity was determined by DXA‐measured body fat percentage (> 25% in men/boys and > 35% in women/girls) [15]. Sarcopenic obesity was defined as the coexistence of excess adiposity and low muscle mass. Furthermore, possible sarcopenia was identified by low muscle strength (handgrip strength < 28 kg in men and < 18 kg in women), whereas sarcopenia was confirmed by the presence of both low muscle mass and low muscle strength [8].

Muscle strength was assessed using a baseline hydraulic hand dynamometer and a pinch gauge (Fabrication Enterprises, USA). Participants were seated with the trunk in the upright position, shoulders and elbows flexed at 90° and wrists in a neutral position without touching the trunk. Participants squeezed with full force for at least 2 s. After one practice trial, two attempts were made per hand alternately, with a 1‐min rest interval. The maximum force (kg) was recorded [17]. Reference values were stratified by age (paediatric norms for 7–17 years old; adult norms for those aged ≥ 18 years) [18, 19]. Physical performance was evaluated using the Short Physical Performance Battery (SPPB), scored from 0 to 12. This included (1) balance test (side‐by‐side, semi‐tandem, full‐tandem for 10 s); (2) gait speed (4‐m walk at normal pace); and (3) five‐time chair stand test (sit‐to‐stand as quickly as possible). Low physical performance was defined as an SPPB score ≤ 9, gait speed < 1.0 m/s or chair stand duration ≥ 12 s [20]. Both the baseline hydraulic hand dynamometer and SPPB are widely recognized as validated tools with high test–retest reliability for the diagnosis of sarcopenia [17, 20].

### 2.7 Statistical Analysis

Data were analysed using SPSS Version 25 (IBM Corp, Armonk, NY, USA). Normality of data distribution was rigorously assessed using the Shapiro–Wilk test. Descriptive statistics for continuous variables are expressed as means ± SDs for normally distributed data, or medians and interquartile ranges (IQRs) for non‐normally distributed variables. Categorical variables are presented as counts and percentages. Homogeneity of variance between groups was verified using Levene's test. Comparisons between subgroups were performed using the independent *t*‐test for parametric data or the Mann–Whitney *U* test for non‐parametric continuous data. For categorical variables, differences between groups were analysed using Pearson's chi‐square test; however, when the expected cell count was < 5 (specifically for the sex hormone therapy variable), Fisher's exact test was employed to ensure statistical validity. Complete case analysis was performed as there were no missing data for the primary outcomes (ASMI and ultrasound parameters). Bivariate correlation analyses were conducted using either Pearson's or Spearman's coefficients depending on the normality of the variables. To identify independent predictors of ASMI, a multiple linear regression analysis was performed. Potential confounding factors—age, sex and BMI—were entered into the model as control variables. Collinearity diagnostics were examined, ensuring variance inflation factor (VIF) values were < 10 and tolerance was within acceptable limits. Finally, the diagnostic utility of the ultrasound parameters for detecting low muscle mass was assessed through receiver operating characteristic (ROC) curve analysis, from which the area under the curve (AUC), sensitivity and specificity were calculated. All statistical tests were two‐sided, and a *p*‐value of < 0.05 was considered statistically significant.

## 3 Results

### 3.1 Recruitment and Participant Characteristics

Fifty‐three individuals with PWS were assessed for eligibility. Five were excluded: three who declined participation due to geographical distance, one who did not meet the inclusion criteria and one who was unable to cooperate. The final cohort comprised 48 participants (52.1% men, *n* = 25) and had a median age of 19.50 years (IQR: 12.25–26.75). Regarding the genetic aetiology, 62.5% (*n* = 30) had the deletion subtype. All participants had a history of growth hormone treatment; 35.4% (*n* = 17) were receiving active growth hormone replacement, and 14.6% (*n* = 7) were on sex hormone therapy at enrolment. Statistical analysis of baseline characteristics revealed no significant differences between male and female participants regarding age, weight, height, BMI or genetic subtype (all *p* > 0.05), indicating a well‐balanced cohort for gender‐based comparisons. The only significant baseline difference was the prevalence of sex hormone therapy (*p* = 0.010). The baseline demographic characteristics of the study participants are presented in Table 1.

**TABLE 1:** Demographic data.

Characteristics	Total (*N* = 48)	Male (*N* = 25)	Female (*N* = 23)	*p* (Male vs. female)
Demographics				
Age (years), median [IQR]	19.5 [12.3–26.8]	19.0 [10.0, 28.0]	20.0 [14.0, 24.0]	0.926^a^
Anthropometrics				
Weight (kg), median [IQR]	62.0 [48.3–77.5]	62.0 [43.0–84.8]	62.0 [49.2–75.5]	0.453^a^
Height (cm), median [IQR]	154 [142–162]	157[139.5–166.5]	150 [142.0–158.0]	0.301^a^
Body mass index (kg/m^2^), median [IQR]	27.1 [22.2–31.4]	25.5 [22.3–31.3]	28.3 [21.7–31.9]	0.628^a^
Gene subtype, *n* (%)				0.709^b^
Deletion	30 (62.5)	15 (60.0)	15 (65.2)	
Non‐deletion	18 (37.5)	10 (40.0)	8 (34.8)	
Comorbid conditions and treatment				
Diabetes mellitus, *n* (%)	11 (22.9)	4 (16.0)	7 (30.4)	0.235^b^
Dyslipidaemia, *n* (%)	13 (27.1)	4 (16.0)	9 (39.1)	0.072^b^
History of growth hormone (GH) use, %	48 (100)	25 (100)	23 (100)	
Current growth hormone therapy, *n* (%)	17 (35.4)	10 (40.0)	7 (30.4)	0.489^b^
Current sex hormone therapy, *n* (%)	7 (14.6)	7 (28.0)	0 (0.0)	**0.010** ^c^

*Note:* Data are presented as *n* (%) for categorical variables and median [interquartile range] for continuous variables. All participants (100%) were of Han Chinese ethnicity and residents of Taiwan. Bold indicates statistical significance (*p* < 0.05). Statistical analysis: *p*‐values represent the statistical comparison between male and female subgroups. No significant demographic or anthropometric differences exists between sexes (*p* > 0.05).

Abbreviations: BMI, body mass index; IQR, interquartile range.

^a^ *p*‐values are calculated using the Mann–Whitney *U* test.

^b^ *p*‐value calculated using Pearson's chi‐square test.

^c^ *p*‐value calculated using Fisher's exact test.

#### 3.1.1 Phenotypic Profile: Body Composition, Muscle Architecture and Functional Capacity

The study population exhibited a distinct phenotype characterized by ubiquitous adiposity and a high prevalence of low muscle function. DXA analysis revealed that 100% (*n* = 48) of the cohort met the criteria for obesity. In terms of muscle mass, the median ASMI was 5.58 kg/m^2^ (IQR: 4.56–6.33). Regarding sarcopenia indicators, 89.6% (*n* = 43) exhibited low muscle strength (possible sarcopenia), whereas 60.4% (*n* = 29) met the criteria for low muscle mass by DXA.

**FIGURE 1:**
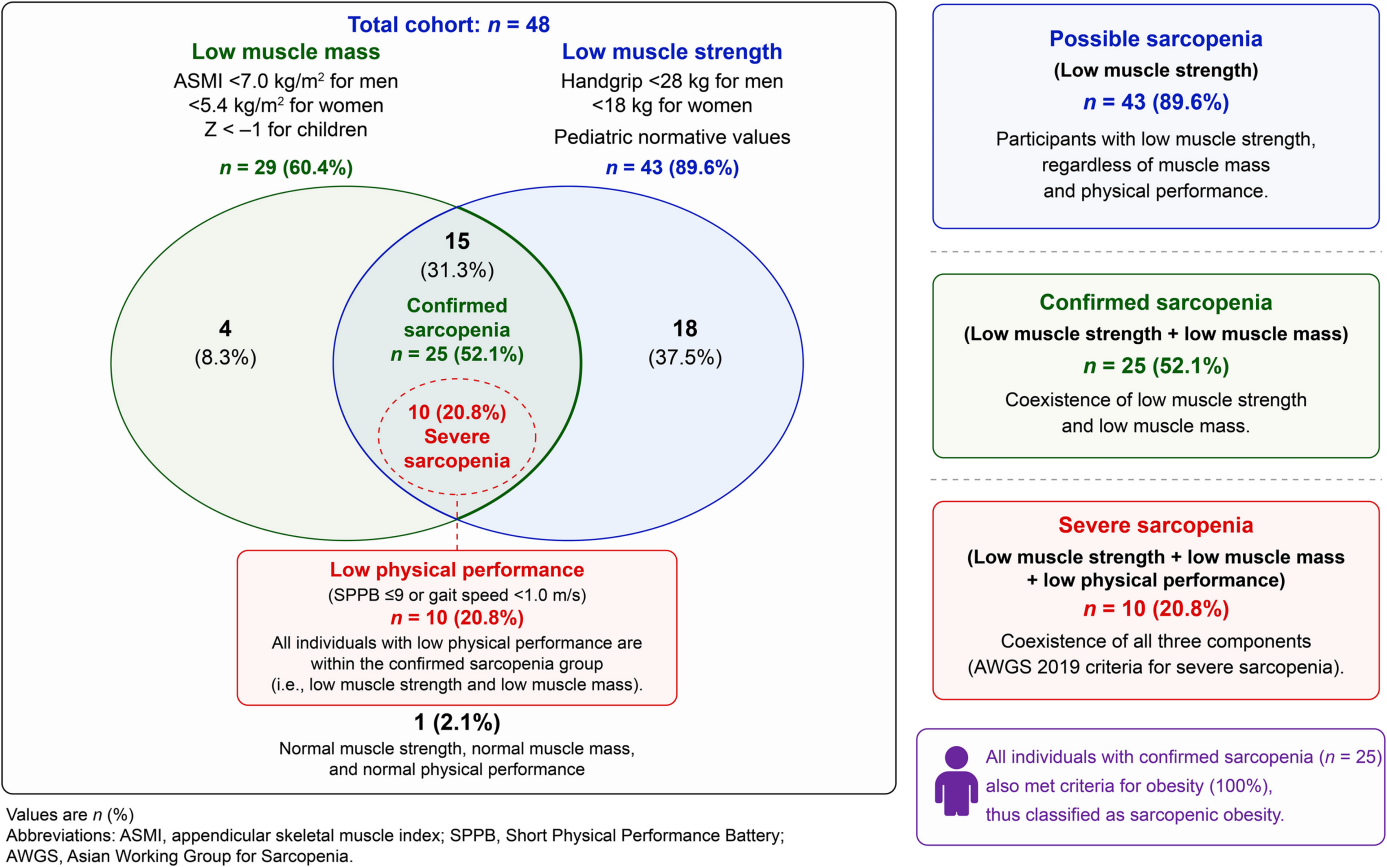
Prevalence and overlap of sarcopenia components in the PWS cohort (*N* = 48). The Venn diagram illustrates the distribution and overlaps of sarcopenia criteria based on AWGS 2019 guidelines. The green circle represents participants with low muscle mass (*n* = 29, 60.4%), and the blue circle represents those with low muscle strength (*n* = 43, 89.6%). The intersection of these two groups identifies individuals with confirmed sarcopenia (*n* = 25, 52.1%). Within the confirmed sarcopenia group, a subset with concurrent low physical performance (red dashed circle, *n* = 10, 20.8%) is classified as having severe sarcopenia. Notably, all participants with low physical performance fell within the confirmed sarcopenia group. Given the 100% prevalence of obesity in the study cohort, the confirmed sarcopenia group (*n* = 25) also represents the prevalence of sarcopenic obesity. One individual (2.1%) exhibited normal muscle mass, strength and physical performance. ASMI, appendicular skeletal muscle index; AWGS, Asian Working Group for Sarcopenia; PWS, Prader–Willi syndrome; SPPB, Short Physical Performance Battery.

When assessing the concordance between strength and mass, 52.1% (*n* = 25) of participants met the criteria for confirmed sarcopenia (coexisting low strength and low mass). Given the universal presence of obesity in this cohort, these 25 individuals were also classified as having sarcopenic obesity. Furthermore, 10 participants (20.8%) demonstrated concurrent low physical performance (SPPB ≤ 9 or gait speed < 1.0 m/s), thus meeting the criteria for severe sarcopenia. Only one participant (2.1%) exhibited a phenotype of normal muscle strength and normal muscle mass. A Venn diagram illustrating the overlap between these components is presented in Figure 1.

Ultrasound assessment indicated a median RF CSA of 7.00 cm^2^ (IQR: 4.42–7.96) and RF MT of 1.71 cm (IQR: 1.51–1.89). The median pennation angles for the vastus lateralis and gastrocnemius medialis were 13.69° and 17.19°, respectively.

Functionally, a discordance was observed between upper and lower limb performance. Upper limb strength was profoundly compromised, with median handgrip strength (14.04 kg) and lateral pinch strength (3.96 kg) consistently below the 10th percentile of age‐matched normative data. In contrast, lower limb functional mobility was relatively preserved, evidenced by a median gait speed of 1.03 m/s and a median SPPB score of 11.0. Table 2 summarizes the muscle characteristics, physical functional capacity and body composition profiles of the study population (*n* = 48).

**TABLE 2:** Results of the muscle ultrasound, physical performance and DXA.

	Total (*N* = 48)	Male (*N* = 25)	Female (*N* = 23)	*p*
Ultrasound parameters				
RF_CSA_ (cm^2^)	7.00 [4.42–7.96]	6.96 [4.26–8.54]	7.04 [4.38–7.73]	0.419^a^
RF muscle thickness (cm)	1.71 [1.51–1.89]	1.79 [1.51–1.95]	1.69 [1.43–1.85]	0.817^a^
VL muscle thickness (cm)	1.99 [1.83–2.32]	2.12 [1.78–2.33]	1.97 [1.84–2.28]	0.621^a^
GM muscle thickness (cm)	1.56 [1.38–1.83]	1.59 [1.38–1.90]	1.49 [1.29–1.69]	0.144^a^
VL pennation angle (°)	13.69 [10.90–17.13]	14.21 [11.11–18.44]	12.31 [10.37–15.36]	0.269^a^
GM pennation angle (°)	17.19 [13.84–22.21]	15.12 [13.37–23.39]	17.78 [14.36–21.33]	0.958^a^
Physical performance				
Grip strength (kg)	14.04 [9.06–19.82]	14.50 [9.06–21.97]	12.68 [9.06–15.40]	0.189^a^
Lateral pinch strength (kg)	3.96 [3.17–5.32]	4.53 [3.17–5.78]	3.40 [2.94–4.53]	0.053^a^
Gait speed (m/s)	1.03 [0.94–1.37]	1.05 [0.97–1.41]	1.03 [0.91–1.34]	0.174^a^
Five‐time chair stand test (s)	11.16 [9.10–12.71]	11.02 [8.44–12.91]	11.26 [9.36–12.36]	0.391^a^
Short Physical Performance Battery	11.0 [10.0–12.0]	11.0 [10.0–12.0]	11.0 [10.0–12.0]	0.656^a^
DXA				
Total lean mass (kg)	31.84 [22.31–37.45]	33.86 [19.89–43.31]	31.53 [26.70–34.56]	0.257^a^
Skeletal muscle mass (kg/m^2^)	13.10 [11.08–15.30]	13.00 [11.00–16.15]	13.40 [11.40–15.00]	0.750^a^
Appendicular skeletal muscle index (kg/m^2^)	5.58 [4.56–6.33]	5.73 [4.56–6.98]	5.55 [4.56–6.19]	0.241^a^
Total fat mass (kg)	28.78 [21.41–38.18]	28.37 [21.52–39.83]	29.57 [21.30–37.78]	0.664^a^
Body fat percentage (%)	45.9 [41.1–48.6]	44.4 [39.5–49.6]	47.9 [44.4–50.7]	0.128^a^
Fat mass index (kg/m^2^)	12.20 [9.40–15.23]	11.60 [8.18–15.40]	12.90 [9.78–15.30]	0.622^a^
Android fat mass (kg)	2.12 [1.39–2.93]	2.05 [1.35–3.57]	2.12 [1.49–2.79]	0.530^a^
Gynoid fat mass (kg)	4.37 [3.33–5.91]	4.21 [3.02–6.23]	4.59 [3.35–5.84]	0.968^a^
Android to gynoid ratio	1.03 [0.94–1.06]	1.04 [0.97–1.09]	0.96 [0.91–1.04]	0.127^a^
VAT, CSA (cm^2^)	101.5 [73.5–157.5]	86.5 [75.2–136.0]	124.0 [70.6–163.0]	0.450^a^

*Note:* Data are presented as median [interquartile range].

Abbreviations: ASMI, appendicular skeletal muscle index; BMI, body mass index; CSA, cross‐sectional area; DXA, dual‐energy x‐ray absorptiometry; GH, growth hormone; GM, gastrocnemius medialis; IQR, interquartile range; MT, muscle thickness; PA, pennation angle; RF, rectus femoris; SPPB, Short Physical Performance Battery; VAT CSA, visceral adipose tissue cross‐sectional area; VL, vastus lateralis.

^a^ *p*‐values are calculated using the Mann–Whitney *U* test.

#### 3.1.2 Associations Between Adiposity, Muscle Architecture and Functional Metrics

Spearman correlation analysis revealed distinct patterns of association between adiposity (FMI), ultrasound metrics and primary sarcopenia indices (Table 3).

**TABLE 3:** Spearman's rank correlation coefficients between ultrasound measurements and body composition, muscle strength and physical performance.

	Ultrasound measurements						Adiposity
	RF CSA	RF MT	VL MT	GM MT	VL PA	GM PA	FMI
Muscle mass (DXA)							
ASMI	0.587**	0.486**	0.559**	0.689**	0.057	0.308*	0.778**
Skeletal muscle mass	0.511**	0.359*	0.427**	0.623**	−0.015	0.323*	0.744**
Adiposity (DXA)							
FMI	0.432**	0.277	0.397**	0.501**	0.009	0.142	1
Muscle strength							
Handgrip strength	0.482**	0.230	0.421**	0.613**	0.004	0.199	0.276
Lateral pinch strength	0.511**	0.271	0.489**	0.532**	0.054	0.165	0.241
Physical performance							
Gait speed	−0.193	−0.306*	−0.168	−0.297*	0.009	−0.040	−0.033
SPPB	0.199	0.277	0.354*	0.127	0.165	0.022	−0.066

*Note:* Values are Spearman's rank correlation coefficients (*ρ*).

Abbreviations: ASMI, appendicular skeletal muscle index; CSA, cross‐sectional area; FMI, fat mass index; GM, gastrocnemius medialis; MT, muscle thickness; PA, pennation angle; RF, rectus femoris; SPPB, Short Physical Performance Battery; VL, vastus lateralis.

* *p* < 0.05.

** *p* < 0.01.

##### 3.1.2.1 Ultrasound and Muscle Mass

**FIGURE 2:**
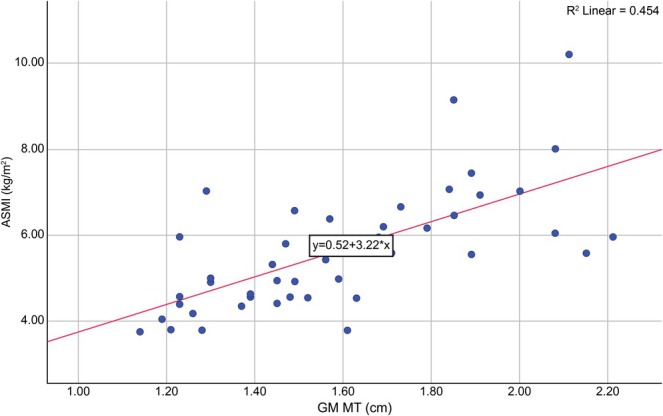
Correlation between appendicular skeletal muscle index (ASMI) and gastrocnemius medialis muscle thickness (GM MT) in the PWS cohort A significant positive correlation was observed (ρ = 0.689, *p* < 0.001). The solid line represents the linear regression fit (*R* ^2^ = 0.847), and the shaded area the 95% confidence interval. ASMI, appendicular skeletal muscle index; GM MT, gastrocnemius medialis muscle thickness; PWS, Prader–Willi syndrome.

GM MT demonstrated the strongest correlation with ASMI (ρ = 0.689, *p* < 0.001, 95% confidence interval [CI] 0.50–0.81), followed by RF CSA (ρ = 0.587, *p* < 0.001, 95% CI 0.36–0.75). Notably, although distal muscle thickness (GM MT) was also strongly associated with handgrip strength (ρ = 0.613, *p* < 0.001, 95% CI 0.40–0.76), proximal measures such as RF MT showed a significantly weaker and non‐significant relationship with strength (ρ = 0.230, *p* > 0.05). Regarding muscle architecture, GM pennation angle showed a weak but significant correlation with ASMI (ρ = 0.308, *p* = 0.033, 95% CI 0.03–0.54), whereas vastus lateralis pennation angle showed no significant associations with physical performance measures. We also analysed functional mobility using the five‐time chair stand test; however, no significant correlations were observed between this test and any ultrasound parameters (data not shown). A scatter plot illustrating the relationship between the primary predictor (GM MT) and ASMI is presented in Figure 2.

##### 3.1.2.2 The Impact of Adiposity: The ‘Loading Paradox’

To evaluate the impact of obesity on the sarcopenia phenotype, we analysed the correlations between FMI and all study parameters (Table 3). A strong positive association was observed between FMI and ASMI (ρ = 0.778, *p* < 0.001), suggesting that excess adiposity acts as a significant stimulus for absolute muscle mass accrual. This ‘loading effect’ was reflected in the ultrasound metrics, where FMI showed a significant positive correlation with GM MT (ρ = 0.501, *p* < 0.01) and RF CSA (ρ = 0.432, *p* < 0.01). However, this relationship was site‐specific; the correlation between FMI and proximal RF MT was notably weaker and did not reach statistical significance (ρ = 0.277, *p* = 0.056). Crucially, despite the strong link between adiposity and muscle mass, a clear dissociation was observed regarding muscle function. FMI showed no significant correlation with physical performance (SPPB: ρ = −0.066, *p* = 0.655) or handgrip strength (ρ = 0.276, *p* = 0.058). This indicates that although increased body mass in PWS is associated with greater muscle quantity, it does not translate into improved muscle quality or functional capacity.

#### 3.1.3 Predictors of Muscle Mass and Diagnostic Utility

To identify independent predictors of ASMI, a multiple linear regression model was established (Table 4). In the final optimized model, BMI was the strongest positive predictor (B = 0.128, *p* < 0.001), indicating that higher BMI was associated with higher ASMI. Age (B = 0.020, *p* = 0.044) and sex (B = −0.376, *p* = 0.029) were also significant predictors. Notably, GM MT remained a significant independent predictor of ASMI (B = 0.957, *p* = 0.011) after adjusting for body size (every 1‐cm increase in GM MT was associated with a 0.957 kg/m^2^ increase in ASMI). Collinearity diagnostics confirmed the model's validity, with all VIF values falling between 1.00 and 1.70.

**TABLE 4:** Multivariable linear regression analysis for independent predictors of ASMI.

Predictor	B (unstandardized)	SE	β (standardized)	*t*	*p*	95% CI (lower, upper)	VIF
(Constant)	0.711	0.574	—	1.239	0.222	−0.447‐1.869	—
Age	0.020	0.010	0.132	2.073	0.044	0.001–0.040	1.129
Sex (female)^a^	−0.376	0.167	−0.140	−2.256	0.029	−0.712–0.040	1.080
BMI (kg/m^2^)	0.128	0.013	0.727	9.903	< 0.001	0.102–0.154	1.513
GM MT (cm)	0.957	0.362	0.201	2.644	0.011	0.227–1.688	1.615

*Note:* Dependent variable: ASMI. Model summary: *R* = 0.920, *R* ^2^ = 0.847, adjusted *R* ^2^ = 0.832. Adjustment for covariates: Age, sex and BMI are included as covariates in the multivariable model to adjust for their potential confounding effects on muscle mass. Predictor context: Although BMI and GM MT serve as independent predictors of reduced skeletal muscle mass (ASMI), this reduction represents the morphological component of the sarcopenia phenotype common in Prader–Willi syndrome. Multicollinearity: All VIF values are < 10, indicating no significant multicollinearity between predictors.

Abbreviations: β, standardized regression coefficient; ASMI, appendicular skeletal muscle index; B, unstandardized regression coefficient; BMI, body mass index; CI, confidence interval; GM MT, gastrocnemius medialis muscle thickness; SE, standard error; VIF, variance inflation factor.

^a^ Sex was coded as male = 1, female = 2; the negative coefficient indicates lower ASMI in females compared to males.

**FIGURE 3:**
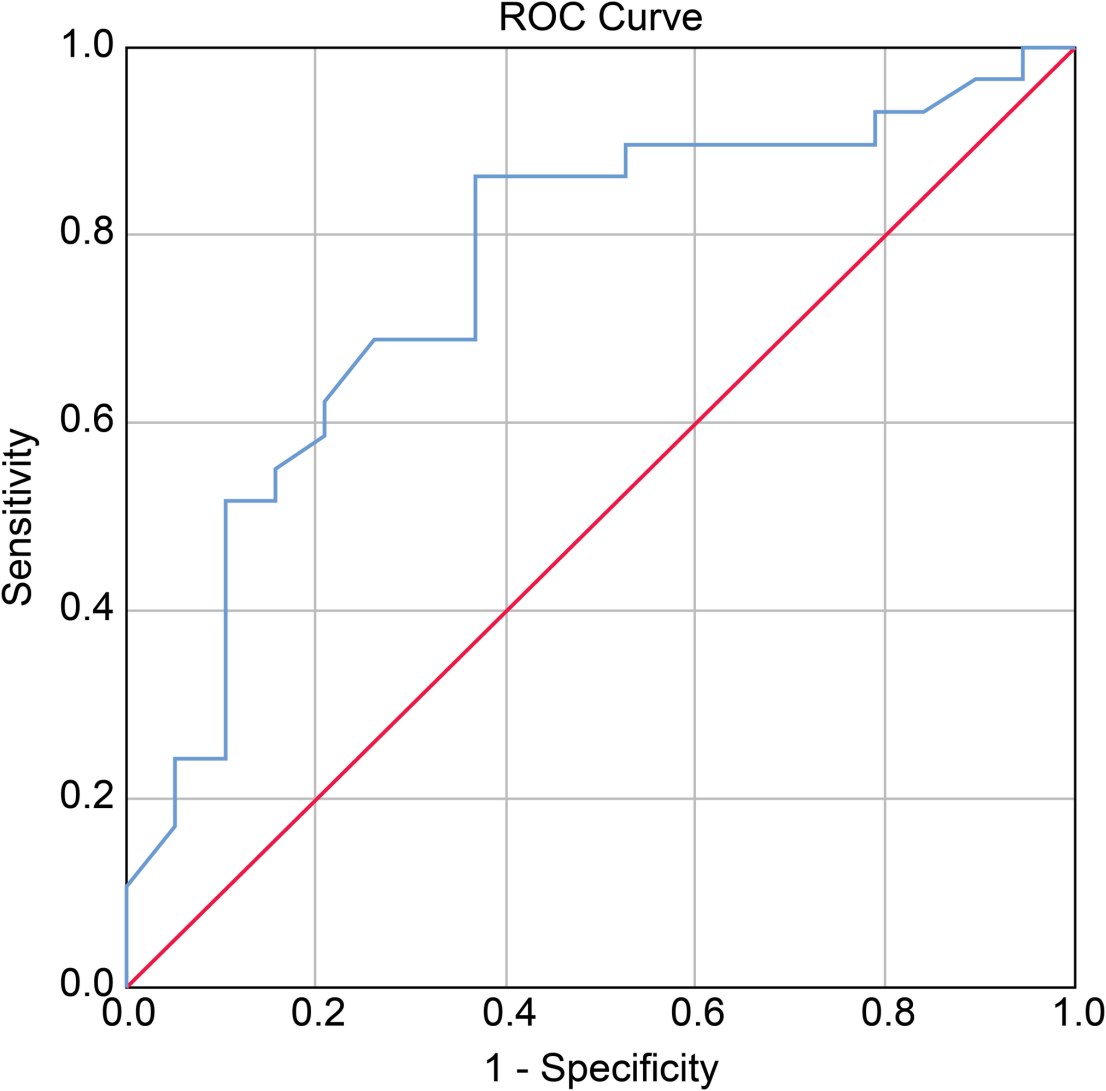
ROC curve analysis of GM MT for the diagnosis of low muscle mass The AUC was 0.759 (*p* = 0.003). The optimal cut‐off value for predicting low muscle mass is 1.69 cm. The diagonal line represents the reference line (AUC = 0.5). AUC, area under the curve; GM MT, gastrocnemius medialis muscle thickness; ROC, receiver operating characteristic.

ROC curve analysis was used to evaluate the utility of GM MT for detecting sarcopenia (Figure 3). This analysis demonstrated good diagnostic accuracy (AUC = 0.759, 95% CI 0.616–0.901, *p* = 0.003). The optimal cut‐off value of 1.69 cm showed a sensitivity of 86.2% and a specificity of 63.2% for detecting low muscle mass.

## 4 Discussion

### 4.1 PWS Phenotype: Sarcopenic Obesity and Functional Discordance

The universal prevalence of obesity (100%) and high rate of confirmed sarcopenia (52.1%) in our cohort underscores that PWS is a quintessential model of sarcopenic obesity. The prevalence of low muscle mass observed here aligns with previous reports indicating that lean mass deficits in PWS persist even in the era of growth hormone therapy [21, 22]. However, a novel observation in our study is the functional divergence between upper and lower extremities. Whereas handgrip strength was universally poor (< 10th percentile), gait speed and SPPB scores were unexpectedly preserved. We hypothesize that this ‘relative preservation’ of the lower limb function is likely a result of the ‘training effect’ imposed by excess body weight. Unlike the upper limbs, which are unloaded, the lower extremity anti‐gravity muscles, specifically the gastrocnemius medialis, are constantly subjected to high mechanical loads during ambulation [10]. Our correlation analysis reveals that although adiposity (FMI) correlated with most lower limb muscle metrics, this relationship was the most robust at the distal site (GM MT: ρ = 0.501, *p* < 0.01) compared to proximal muscle thickness (RF MT: ρ = 0.277, *p* > 0.05; Table 3). This gradient implies that the mechanical loading effect of excess weight is most prominently reflected in the distal anti‐gravity musculature. This further suggests that in individuals with PWS, excess fat mass may paradoxically serve as a chronic mechanical stimulus that facilitates the accrual or preservation of weight‐bearing muscle tissue. This observation aligns with the ‘muscle–bone unit’ concept proposed by Fintini et al., where increased body mass exerts a stimulus that promotes the development of cortical bone as a ‘kind of armor’ for the body [5]. This process suggests the presence of a robust ‘muscle–bone unit’ where consistent weight‐bearing activity produces soluble signals that result in increased lean mass and turnover while counteracting the accumulation of intramuscular fat [5]. This chronic loading may induce a compensatory adaptation that maintains functional mobility despite intrinsic muscle quality deficits, a phenomenon previously described in non‐PWS obesity but rarely quantified in the population with PWS [23]. Crucially, our findings regarding the preservation of the gastrocnemius medialis suggest that distal anti‐gravity muscles are strongly representative of the functional mass maintained by the daily mechanical demands of carrying excess weight. Consequently, measuring the thickness of these distal muscles provides a more accurate diagnostic reflection of the working muscle mass in the PWS population than proximal sites, which are more prone to measurement error from subcutaneous adiposity and myosteatosis.

### 4.2 Diagnostic Utility of Gastrocnemius Ultrasound

A key contribution of this study is the validation of the gastrocnemius medialis as a superior predictive site compared to the rectus femoris or vastus lateralis. In general sarcopenia literature, the rectus femoris is often the preferred site for ultrasound assessment [9]. However, our multivariable regression showed that GM MT was the strongest independent predictor of ASMI. We posit that in individuals with PWS, the calf muscle may be a more reliable anatomical marker than the thigh for two reasons. First, the excessive subcutaneous adipose tissue in the thigh region of patients with PWS can attenuate ultrasound beam penetration and complicate fascial border identification, potentially introducing measurement error in the rectus femoris/vastus lateralis assessment [6]. The gastrocnemius medialis, being relatively more superficial and distal, may offer clearer imaging. Second, given the preserved ambulatory capacity observed in our cohort, the gastrocnemius medialis plays a critical biomechanical role in propulsion; thus, its morphology may better reflect the ‘functional’ muscle mass maintained by daily activity than the thigh muscles.

### 4.3 Muscle Thickness vs. Pennation Angle

Whereas GM MT was a strong predictor, pennation angle showed weaker associations and was not significant in the final model. Muscle thickness is a proxy for muscle size, whereas pennation angle reflects fibre packing and potential force‐generating capacity. In PWS, the primary deficit appears to be absolute hypoplasia (lack of size) rather than architectural remodelling [24]. It is also possible that intramuscular fat infiltration (myosteatosis), which is common in PWS, obscures the fibre striations needed to accurately measure pennation angle, making muscle thickness a more reproducible and robust metric for this specific population [25]. This is consistent with the musculoskeletal environment in obesity, where multipotent mesenchymal stem cells are driven towards adipocyte differentiation at the expense of osteoblast and myoblast lineages [5]. This cellular imbalance is further exacerbated by the release of pro‐inflammatory cytokines, such as tumour necrosis factor‐α and interleukin‐6, from bone marrow and adipose tissue, which alter the local microenvironment and promote tissue fragility [5].

### 4.4 Clinical Significance and Practical Applications

The results of this study have important implications for the multidisciplinary management of PWS. For clinicians, the validation of the gastrocnemius medialis as a predictive site provides a practical, radiation‐free bedside tool that bypasses the physical weight capacity constraints and behavioural challenges often associated with DXA in this population. For researchers, our findings establish a reliable protocol for assessing muscle mass in populations with high proximal adiposity, suggesting that distal anti‐gravity muscles may serve as a superior proxy for ‘working’ muscle mass. For patients and caregivers, the integration of ultrasound into routine clinical monitoring offers a non‐invasive means to track the efficacy of growth hormone therapy and exercise interventions, ultimately supporting the maintenance of functional independence and metabolic health.

### 4.5 Strengths and Limitations

This study has several strengths, including the use of gold‐standard DXA for body composition, strict inclusion criteria to ensure a homogenous genetic and environmental background (all participants were of Han Chinese ethnicity and resided in Taiwan) and a high statistical power (0.99) despite disease rarity. However, limitations must also be acknowledged. First, the cross‐sectional design precludes causal inferences regarding the progression of sarcopenia over time. Whereas sarcopenia definition was originally developed for older adults, PWS represents a unique congenital model of sarcopenia characterized by muscle mass and strength deficits persisting from infancy through adulthood. Consequently, identifying these components early is clinically relevant, as these motor deficits significantly impact caregiver quality of life and functional independence. Furthermore, although it remains unclear how the progression from possible sarcopenia to sarcopenic obesity affects long‐term longevity, our team has an ongoing 5‐year longitudinal follow‐up study (projected completion February 2026) to evaluate these developmental trajectories. Second, due to the lack of PWS‐specific reference standards for muscle ultrasound, we relied on the general population norms and age‐matched controls. Although this is a standard approach in rare disease research, it may not fully capture the unique physiological baseline of PWS. Finally, although we controlled for key confounders, we did not objectively measure the daily physical activity levels (e.g., using accelerometers), which could partially explain the variance in lower limb muscle preservation.

## 5 Conclusions

This study demonstrates that the GM MT is a robust and independent predictor of ASMI in individuals with PWS. Our findings reveal a distinct phenotype in this population characterized by ubiquitous obesity and a high prevalence of sarcopenia (52.1%). Whereas upper limb strength was profoundly compromised, lower limb functional mobility remained relatively preserved. Crucially, GM MT showed significant diagnostic utility (AUC = 0.759) for identifying low muscle mass, with an optimal cut‐off value of 1.69 cm. Consequently, we recommend integrating gastrocnemius medialis ultrasound assessment, specifically of the calf muscle, alongside standard anthropometric and demographic indices, as a practical, radiation‐free strategy to screen for sarcopenia and monitor muscle health in the PWS population.

## Funding

This study was supported by grants from the Taipei Tzu Chi Hospital, Buddhist Tzu Chi Medical Foundation (Grant numbers TCRD‐TPE‐112‐28 and TCRD‐TPE‐112‐RT‐5). The funding sources had no role in the study design; the collection, analysis or interpretation of data; or the decision to submit the manuscript for publication.

## Ethics Statement

The study was approved by the Taipei Tzu Chi Hospital, Buddhist Tzu Chi Medical Foundation Institutional Review Board (11‐XD‐082), and registered in Clinical Trials (NCT06448871). All participants and/or their legal guardians provided written informed consent prior to enrolment.

## Conflicts of Interest

The authors declare no conflicts of interest.
